# Alpha-Synuclein: From Early Synaptic Dysfunction to Neurodegeneration

**DOI:** 10.3389/fneur.2018.00295

**Published:** 2018-05-04

**Authors:** Veronica Ghiglieri, Valeria Calabrese, Paolo Calabresi

**Affiliations:** ^1^Dipartimento di Filosofia, Scienze Sociali, Umane e della Formazione, Università degli Studi di Perugia, Perugia, Italy; ^2^Laboratorio di Neurofisiologia, Fondazione Santa Lucia, IRCCS, Rome, Italy; ^3^Clinica Neurologica, Dipartimento di Medicina, Università degli Studi di Perugia, Ospedale Santa Maria della Misericordia di Perugia, Perugia, Italy

**Keywords:** synucleinopathy, experimental parkinsonism, neurodegeneration, synaptic plasticity, protein aggregation

## Abstract

Over the last two decades, many experimental and clinical studies have provided solid evidence that alpha-synuclein (α-syn), a small, natively unfolded protein, is closely related to Parkinson’s disease (PD) pathology. To provide an overview on the different roles of this protein, here we propose a synopsis of seminal and recent studies that explored the many aspects of α-syn. Ranging from the physiological functions to its neurodegenerative potential, the relationship with the possible pathogenesis of PD will be discussed. Close attention will be paid on early cellular and molecular alterations associated with the presence of α-syn aggregates.

## The Many Roles of Alpha-Synuclein (α-syn)

Alpha-synuclein is a 140 aminoacid protein, encoded by the *SNCA* gene on human chromosome 4. This protein is mainly expressed in presynaptic sites at several neurotransmitter systems in the central nervous system (CNS) (1). Despite its ubiquitous distribution through many areas involved in complex behaviors, α-syn pathology does not impact on all brain sites of expression, but rather shows a prevalent effect in selective vulnerable sites (1, 2). Moreover, α-syn is highly present in red blood cells (3) and in other extra CNS tissues (4, 5), indicating a wide range of actions of this protein throughout the body.

Although α-syn is gaining increasing consideration as a critical factor in Parkinson’s disease (PD) pathophysiology and 20 years of research have been spent in the attempt to unravel the physiological roles of this protein, its mechanisms of action are still unclear and so are the complex dynamics that characterize its flexibility to adapt and the tendency to become toxic.

α-syn exists in a dynamic balance between monomeric and oligomeric states, which are not easily prone to form fibrils in physiological conditions. Interestingly, its structure predicts the multifunctional properties that have been attributed to this protein (6). As a result, this structural flexibility allows α-syn to adopt a wide range of conformations depending on the environment and binding partners (7, 8). In fact, α-syn can either relate to intracellular and membrane proteins with its enzymatic activity or interact with lipid surfaces and organize membrane activities through steric mechanisms.

Given its prevalent localization at presynaptic sites, the first function described for α-syn was its chaperone function and in particular its ability in controlling exocytosis through management of synaptic vesicle pool and trafficking. Accordingly, mutations of the SNCA gene coding for α-syn leads to functional alterations of SNAP REceptor (SNARE) proteins, a family of receptors that binds the soluble *N*-ethylmaleimide sensitive fusion attachment proteins (SNAP) receptor (SNARE) proteins and regulates their assembly (9). Another presynaptic target for α-syn is the DA active trasporter (DAT) (10, 11).

Upon interaction with lipidic surfaces α-syn binding causes the formation of an amphipathic alpha-helix that in physiologic conditions does not cross the bilayer. Under specific stimulations, oligomers of α-syn may form membrane pores that may dissipate the transmembrane potential, dysregulating ion gradients (12, 13).

Several strategies exist to ensure the prevention of α-syn oligomerization (14–17), including complex hydrophobic interactions between C- and N-tails of the protein (16, 18, 19). Interestingly, α-syn possesses a polar C terminal tail able to interact with the hydrophobic region of a separate denatured protein, sharing structural and functional homology with other molecular chaperones. Thus, the extreme flexibility of this protein also relies on the ability of α-syn to auto assemble and act as an intramolecular chaperone (20). In agreement, α-syn truncated at the C-terminus lacks this auto-chaperone property (21) and aggregates at an increased rate compared with the full-length counterpart (14, 15, 21, 22). Despite its crucial contribution to ensure a good orchestration of processes at the active zones, α-syn translocates late to the terminals during development (23) and its absence seems to be not detrimental for synaptogenesis, indicating that its function is rather essential for stressful and sustained activity over time during the long life of a neuron (24, 25). All these characteristics strongly argue for a critical role in neurotransmitter release and synaptic plasticity. A feature that makes multifunctional α-syn as much enigmatic as difficult to counteract in pathological settings is that, like many other disease-associated misfolding proteins, its absence is less detrimental than its accumulation (26, 27). In physiological conditions, during proteins translation, polypeptides fold under control of chaperones. Errors in assembly are frequent and become more common with aging but they are usually limited by several quality control mechanisms that target denatured and misfolded proteins to degradation (28–30). Given the complex management of this protein expression and the high versatility of its functions, failures in these homeostatic steps do not simply bring to an abnormal gain of function but rather to a potent trigger for a series of neurodegenerative cascades in the intracellular environment. The possibility that residual physiological functions and compensative mechanisms are in act during degeneration, complicates therapeutic approaches and adds unpredictability to possible manipulation of α-syn functions.

## α-syn Oligomers and Fibrillary Aggregates: Pathological Implications

An increasing body of evidence from studies carried out in animal models and in patients support the hypothesis that the processes underlying α-syn proteostasis have central roles in the pathogenesis of PD. This concept dates back to 20 years ago when two discoveries provided support for a role of possible link between α-syn mutations and PD. The first report was the identification of a missense mutation of this gene (31) causing a form of early-onset familial PD by Polymeropoulos and his research team. In the same year, Spillantini’s group provided experimental evidence that α-syn is the primary structural component of Lewy bodies (LB), intracytoplasmatic inclusions of α-syn aggregates, which are considered the main pathological hallmark of PD (32). Shortly after, also sporadic idiopathic forms of PD were found associated with the presence of LB in the brain parenchyma (33).

In the last years, physiological and pathological functions of α-syn and other misfolding proteins have been investigated in relation with other known aspects of the disease, to explore possible causal relationships. For PD, many risk factors have been identified that include both environmental and genetic causes. Oxidative stress, mitochondrial dysfunctions, neuroinflammation, point mutations, multiplications, and specific polymorphisms are genetic determinants that may cooperate to create ideal conditions for developing PD.

Interestingly, these factors are also determinants that impact on the predisposition of α-syn to exert toxicity.

Despite the existence of redundant quality control systems to ensure a correct assembly of α-syn and the ability of other synucleins to inhibit and control oligomerization of α-syn, this protein may express its neurotoxic potential when soluble monomers initially form oligomers, then progressively combine to form small protofibrils and further aggregate in large, insoluble α-syn fibrils forming LB (34, 35). Although its natural propensity to balance between a soluble and membrane-bound state and its plasticity of conformation, acute triggers of accumulation and aggregation of α-syn can be manifold like overproduction of the protein, failure in the molecular system that cleave misfolded forms, exposure to pH changes, oxidative stress, and mitochondrial overwork.

More chance for aggregation is offered by a variety of post-translational covalent modifications (8) potentially promoting conformational changes that make α-syn more prone to aggregation. For example, tyrosine nitration (Tyr125) and truncation of α-syn at the C-terminus are frequently found in α-syn pathological aggregates and have been shown to promote fibrillation *in vitro* (36, 37).

Finally, a progressive, age-related decline of efficiency in the in proteolytic mechanisms might play a synergistic role in the accumulation of α-syn (38, 39). These observations are consistent with data showing increased levels of α-syn in nigral dopaminergic neurons during normal aging (40).

In the healthy brain, intracellular homeostasis of α-syn is ensured by the combined actions of the ubiquitin–proteasome (UP) system and the lysosomal autophagy system (LAS) with the latter more involved in the clearance of oligomeric assemblies (38). Any failure in these systems is a potential trigger to overproduction and accumulation of α-syn forms, although compensatory mechanisms and additional proteases can take control over the protein maturation (38, 41). An aspect that complicates the scenario is that accumulation of α-syn may itself inhibit these homeostatic systems (42, 43) and reduce chaperoning of misfolded forms, enrolling the whole compartment into a vicious cycle that rapidly and uncontrollably triggers multiple neurodegeneration pathways. Accordingly, several mutations associated with genetic forms of PD are associated with reduced LAS function.

Analysis of LB has been indicative of the post-translational modifications mostly associated with pathogenic forms of α-syn (44). Among them, phosphorylation is probably the most studied modification since Ser129 phosphorylated α-syn is thought to be the dominant form of α-syn in LB (45). In support of this prevalence, a recent proteomics study quantified cortical expression levels of various α-syn forms from PD cases and controls (46).

It remains unclear, however, whether phosphorylation of α-syn impacts the fibrillation process (47). The role of nitration and oxidation in favoring toxic species is more clearly demonstrated in decreasing the tendency of α-syn to form fibrils and stabilizing oligomers, leading to enhanced toxicity (48, 49). Nitration of α-syn at specific residues has been characterized in brains from patients with synucleinopathies (50). Oxidized α-syn may result by way of oxidized derivatives of DA leading to a decrease in fibril formation and a subsequent increase in protofibril accumulation (51).

Truncated α-syn species have been found in LB associated with an increased tendency to form fibrils *in vitro* and with increased toxicity in overexpressing laboratory animals (52, 53) even if evidence of correlation with human disease are scarce (46).

The pathological relevance of α-syn species is extensively debated (44) and stabilization of the amyloid pathway is a main focus of research. It has been proposed that toxic species could be either amyloid-like insoluble fibrils, as the ones found in LB, although more evidence would support a key role for soluble oligomers or protofibrils (35). Several groups have investigated the different states of α-syn aggregation and thoroughly examined the functional consequences of aggregate-associated toxicity producing conflicting results (35, 44). However, the general concept is that α-syn exists under various conformational shapes and oligomeric states in a dynamic balance, modulated by factors either accelerating or inhibiting fibril formation. Genetic mutations related to PD have a role in determining the pattern of expression of the various aggregates (54–56), although the identification and characterization of the toxic α-syn species remain incomplete.

Strategies to counteract α-syn toxicity range from increasing protein clearance, which might be enhanced by stimulating autophagy, to act on α-syn post-translational modifications (44). Also, approaches targeting α-syn aggregation with inhibitors (57–59) or by inducing either passive or active immunization against α-syn species have shown promises in several transgenic mouse models of PD (60–62).

However, limits of this approach have been recently discussed (44). Given the incomplete knowledge of possible cellular roles of oligomers and the many functions covered by this protein, the precise α-syn species to target remains unclear. It is possible to hypothesize that modest presence of specific α-syn aggregates can be useful for the cell as part of compensative mechanism that is still unclear, and that their elimination could be harmful and accelerate instead of counteracting the disease process.

## DA Neurons Vulnerability to α-syn

Relevant to the impact of protein misfolding in brain functions, an aspect that still puzzles researchers in the field of neurodegenerative diseases is the selective vulnerability of certain population of neurons to a wide range of insults.

In PD, dopaminergic neurons of the substantia nigra pars compacta (SNc) show selective neurodegeneration and cell death with reduction of dopamine (DA) levels in the striatum and impairment of several basal ganglia functions. The mechanism by which α-syn injures dopaminergic neurons remains to be fully established.

Alpha-synuclein is related to DA neurons for its ability to modulate DA homeostasis in synapses and to bind and influence the activity of DAT (63–65), although the implicated mechanisms are still debated (66–68). This protein is also an important modulator of DA metabolism as it controls DA synthesis by reducing the phosphorylation state of tyrosine hydroxylase and stabilizing it in its inactive state (69). Accordingly, absence of α-syn exerts considerable impact on the dopaminergic system because it causes decreased striatal DA levels and reduced DAT function (70). Lack of α-syn is also associated with decreased DA striatal uptake (71), reduced number of TH-positive terminals as well as of nigral DA cells (72).

However, the sensitivity of DA neurons to α-syn toxicity does not only depend on the possible lack of support to DA metabolism, but on the intrinsic and selective vulnerability of these neurons to excitotoxic challenge.

A recent review discusses the common traits of neurons of SNc neurons and other nuclei most vulnerable to PD pathology, offering an interesting point of view (73). The authors posit that SNc neurons share particular vulnerability to oxidative stress with cells of other brain nuclei involved in arousal responses and in the control of sensorimotor networks, needed for surviving behaviors such as vigilance, escape, and attack. SNc DA neurons possess at least two characteristics that make them particularly vulnerable to excitotoxic insult.

First, these neurons display an extensive length of branched axons that offer a high number of transmitter release sites. This diffuse axonal arbor might be functional to the coordination of the activity in spatially distributed networks, such as the basal ganglia. However, mitochondrial stress is elevated in the axons of SNc DA neurons and this is one reason why these neurons show increased vulnerability.

Second, DA neurons also have spontaneous activity and act as autonomous pacemakers. Their activity is characterized by large oscillations in intracellular calcium (Ca^2+^) concentration that are driven by the opening of voltage-dependent Cav1 Ca^2+^ channels (also known as L-type Ca^2+^ channels) to ensure a rhythmic (2–10 Hz) spiking (74–76). This ability is associated to low intrinsic Ca^2+^ buffering and requires a strict control of Ca-mediated processes from intracellular stores, promoting Ca^2+^ entry into the mitochondria (77, 78) as well as oxidative phosphorylation and production of ATP (79). All these events are needed to fulfill bioenergetic needs (79, 80) and to avoid undesired compensative activation of ATP-sensitive potassium channels, which would silence ongoing neuronal activity.

Substantia nigra pars compacta cells and other neurons of brain nuclei involved in sensorimotor integration are endowed with this complex set of feedforward control mechanisms that ensure to rapidly implement a correct strategy in response to environmental challenge. A price for this adaptive ability is the vulnerability of the system to age, genetic mutations, or environmental toxins that may increase production of reactive oxygen species that can impair proteostasis, cause accumulation DNA damages, particularly in mitochondria. When mitochondrial dysfunction reaches a level in which mitophagy is impaired, also cellular autophagic processes are affected and UP and LAS systems are compromised. Accordingly, in rodents, SNc, locus coeruleus, and dorsal motor nucleus of the vagus neurons (which are the only ones that have been studied at this level) manifest a basal mitochondrial oxidant stress in the somatodendritic region that is attributable to the feedforward control of oxidative phosphorylation of ATP (78, 81–83). On this line, a recent paper by Burbulla and colleagues (84) analyzed the synergistic detrimental effect of increased levels of α-syn, dopaminergic receptor stimulation, and mitochondrial dysfunction in mice showing functional inactivation of DJ-1, modeling an early-onset genetic form of PD. Interestingly, mice with both DA neuron-specific overexpression of human α-syn A53T (85) and constitutive DJ-1 deficiency show increased levels of oxidized DA in nigral neurons and decreased lysosomal activity compared with mice bearing the single DJ-1 mutation.

All these data support the concept that α-syn induces exacerbation of a Ca^2+^ dyshomeostasis in DA neurons. The paper by Luo and coworkers provided experimental evidence for this link by studying the potential effects of increased α-syn levels on processes downstream of the Ca^2+^-signaling pathway, demonstrating the contribution of a new calcium-dependent pathway in the dopaminergic neuronal loss (86). A possible explanation resides in the combination of the α-syn oligomers property to trigger Ca^2+^ influx and the intrinsic physiological characteristics of DA neurons. This neuronal population is in fact characterized by pacemaker activity that, as described above, depends on a complex homeostatic regulation, which involves the activity of L-type calcium channels (87), bringing the DA neuron on the edge of triggering neurodegenerative pathways. Another study that has been instrumental in deciphering the link between DA neurons and α-syn is the paper by Feng et al. (88) demonstrating that in particular conditions, like overexpression of wild-type (WT) α-syn, oligomers causes the formation of pore-like structures throughout the membrane acting as non-selective channels. This was associated with increase in membrane conductance and with cell death (88).

## Beyond the Braak Hypothesis

The prevalent belief on the progression of PD neurodegeneration is based on the observation of an ascending pattern of its clinical manifestations that identifies the disease phase. The idea is that toxic species of α-syn progressively reach more brain regions over the course of the disease, as suggested by Braak and coworkers (89), starting from peripheral body dysfunctions and olfactory impairment through central brainstem functions to end with alterations of higher functions over years or decades following the first exposure to stressors.

In this theoretical framework, the speculation that prodromal symptoms of PD (hyposmia, constipation, and autonomic dysfunctions) might be due to peripheral seeding of α-syn aggregates gained a broad consideration in the field. Indeed, in prodromal phases, inflammation in the gastrointestinal tract or in the olfactory system may trigger the formation of α-syn aggregates (90). This concept is supported by recent evidence obtained in *Snca*-overexpressing mice suggesting a role of gut microbiota in hosting immune and inflammation response linked to α-syn pathology, associated with motor deficits (91).

Thus, α-syn would be released into the synaptic cleft, endocytosed by neighboring neurons, and seed aggregation of endogenous α-syn once inside their new cellular host (92–94). However, this is a much-debated issue and, although many studies support the notion of a spreading of α-syn pathology through a prion-like activity, recent analyses have challenged this theory demonstrating that the distribution of pathology in the brains of PD patients is not consistent with this model. The prion-like nature of α-syn was postulated around a decade ago after the observation of the development of LB-like intracellular inclusions in grafted DA neurons of PD patients who received a transplantation of embryonic mesencephalic grafts 11, 14, and 16 years earlier (95–97). The hypothesis of a “host-to-the-graft” transmission of LB led to the concept that α-syn oligomers may spread from cell to cell through axonal transport and exocytosis, aggregate into LB, and then transferred to other neurons.

A recent study by Peelaerts and colleagues investigated whether different forms of α-syn aggregates are genuine protein strains with a given role and a specific impact on animal physiology (98), based on the hypothesis that different strains could account for the different clinicopathological traits within synucleinopathies (99, 100). The authors propose that α-syn exists and exerts its detrimental effects, in different strains leading to different aggregates that cause as many distinct synucleinopathies (PD, dementia with LB, multiple system atrophy) (98, 101). The most relevant insights from this study are that (1) the dynamic nature of α-syn species is reflected into distinct competencies in the various species that could account for different phenotypes; (2) α-syn strains amplify *in vivo*; and (3) α-syn assemblies cross the blood–brain barrier after intravenous injection.

Although findings in support of the prion-like hypothesis are numerous, an increasing number of studies have recently challenged this vision. Two interesting reviews thoroughly discuss limits of the data collected in support of the ascending theory of α-syn pathology rather supporting a threshold theory to explain controversial data. One of the most important point of the “threshold theory” (102) stems from the simple consideration of PD as a global systemic disease supported by many genetic, cellular, and functional data. The fact that invalidates the ascending theory regards the evidence that brainstem and peripheral neurons are more resistant to insults and less prone to neurodegeneration compared to DA neurons (103–105) and capable of regeneration (106, 107). One explanation of the early dysfunction of brainstem and enteric neurons is due to their low threshold of functional reserve in contrast with the resilience of central neurons as part of widespread interconnected networks that ensure a good degree of compensation and redundancy to conserve higher functions (108, 109). Indeed, central networks have a great ability to compensate for an impaired function of a given central brain area, resulting in a late appearance of motor and cognitive symptoms. The authors propose that parallel pathological events in PD occur at similar rates resulting in the first symptoms pertaining to a peripheral nervous system alteration, due to an earlier functional threshold in the autonomic nervous system compared with midbrain dopaminergic circuitry. This threshold function explains the progression of early symptoms in PD.

## Inflammation and Immune Response in PD

In many disorders of the CNS, a key aspect of neurodegeneration is neuroinflammation. In PD, abnormal functions of astrocytes and increase in soluble inflammatory cytokines from microglia and immune cells have been proposed as a critical player that together with glutamate-mediated excitotoxicity becomes major determinants for pathophysiology. In nigrostriatal degeneration, inflammatory response is invariably associated with α-syn-mediated events. Glial cells, which cover a wide range of functions in support of neuron development, maintenance, and survival, seem to be critically involved at many levels in the neurodegenerative spreading of the disease pathology. These studies have provided evidence supporting CNS immune resident cells role in PD (110). Activation of some glial components, such as astrocytes, however, is not limited to final phases of the inflammation process but it has been recently supposed to play a relevant part in initiating the pathology (111). Conversely, although it is well known that microglia plays a role in abnormal plasticity by its ability to produce inflammation mediators (112), it is less clear if microgliosis is instrumental to disease pathogenesis or a secondary event following the ongoing neurodegeneration and a primary role remains to be defined (113).

Functionally, all neuronal activities require an intact glial function provided by both astrocytes and microglia, which become essential for neurons enrolled into intense synaptic activity, such as DA SNc neurons. Astrocytes, besides their role as structural, metabolic, and trophic support, are directly involved in synaptic transmission, ensuring a proper communication, and avoiding abnormal stimulation of extrasynaptic receptors. While astrocytes can be considered an essential component of an operational synaptic surveillance, microglia is in charge of the immune surveillance in the brain. Abnormal microglia activation was found in autopsy brain tissues from PD patients and in experimental parkinsonism (110, 114–117) and many recent papers have focused on the roles of microglia in PD pathology [reviewed in Ref. (113)] bringing support to the notion that neurodegenerative processes and inflammation coexist and cooperate at the same time to respond to brain insults, and that these events do not just occur in series as the disease progresses. For example, when an immune response is initiated by microglia, astrocytes surround the area, creating a barrier to prevent the spread of toxic signals into the surrounding tissue (118). Neuroinflammation and immune response, including autoimmune activity, share molecular pathways initiated by cellular elements during degeneration.

A recent review paper by Booth and colleagues (111) has provided an extensive overview of the studies that link astrocytes alterations and PD, with particular attention to the monogenic forms of disease in which genetic mutations affect the functions of both principal neurons and astrocytes. A recent transcriptome study demonstrated that of 17 genes that have been implicated in PD, 8 are also expressed in astrocytes and are essential for their homeostasis (119). Although SNCA gene shows a low astrocytic expression, it has been suggested that even a modest presence of α-syn might be challenging for their function. In fact, α-syn initiates and regulates astrocyte activation in response to inflammatory stimuli. Also, astrocytes have been reported to take up aggregated alpha syn. The most fascinating aspect of astrocyte involvement in neuronal degeneration, relevant to PD, is that astrocytes change in shape and function to provide support to DA neurons under intense stressful conditions (120–122). Astrocytes are also able to take up circulating DA precursor l-DOPA to release DA (123), as they express enzymes and the complete machinery for its metabolism, suggesting a close relationship to DA systems.

It has been recently shown that in 6-hydroxydopamine-lesioned rats, modeling late stage PD, marked astrocytosis and microglial activation accompany neurodegeneration over time as the damage progresses, being strikingly visible in striatal samples 2 months after the lesion. Interestingly, following a repetitive transcranial magnetic stimulation (TMS) treatment, reduction of intense astrogliosis and microgliosis was associated with, and may underlie, recovery of corticostriatal plasticity. Such TMS-mediated recovery of glial morphology and function was associated with selective increase of DA in dorsolateral striatum of treated parkinsonian animals (124).

These data are in agreement with studies showing that microglia is implicated in the production of neurotrophins, interleukins, proinflammatory, and antiinflammatory cytokines (114–117) and with studies linking TMS beneficial effects with stabilization of microglia, reduction of neuroinflammation biomarkers (110, 125). Midbrain DA neurons, α-syn, and immune response are also linked together by their involvement in altered Ca^2+^ homeostasis. The paper by Luo et al. (86) demonstrated that in DA neurons of A53T α-syn transgenic mice dysregulation of intracellular Ca^2+^ activates the calcineurin pathway that, in turn, increases the translocation rate of the nuclear factor of activated T cells (NFAT) from cytosolic to nuclear compartments. This is associated with the expression of cytokine genes, in human T cells and enhanced cell death in SNc. Inhibition of calcineurin renormalizes the mitochondrial Ca^2+^ fluxes rescuing the α-syn-induced loss of primary mesencephalic DA neuron cultures.

In support of the relationships between immune system and α-syn pathology, an interesting study reported significantly higher service levels of antibodies against monomeric α-syn in patients at early phases of disease. This paper postulates that autoimmunity responses take part in a compensative attempt of neuroprotection (126). A recent paper by Shalash and collaborators has shown that α-syn autoantibodies (AIAs) can be a promising avenue in the field of peripheral biomarkers compared patients with PD, to patients with AD along with controls (127). These molecules are also produced in gender-dependent fashion, across the lifespan during the development and can be detected in healthy young subjects, with titers similar to healthy adults (128). This might suggest that autoantibodies production might be optimized over time in response to environmental stimuli.

A series of studies by Sulzer and coworkers further supports the existence of a link between α-syn and immune response. During PD neuroinflammation, the blood–brain barrier becomes permeable to immune cells recruited into the CNS by massive proinflammatory cytokine production from microglia (129, 130). A recent paper by this group shows that this process triggers an immune response against identified α-syn epitopes in PD patients who presents specific major histocompatibility complex alleles. In particular, two antigenic regions have been identified: the first near the N terminus (called Y39 region) and the second near the C terminus, the well-known S129 region, containing the amino acid residue S129, whose phosphorylation has been associated with α-syn pathology (131).

## Early Synaptic Alterations Preceding Neurodegeneration

Although many advances have been made in deciphering the mechanisms by which α-syn triggers neurodegenerative pathways, the ability of mammalian brain to compensate for loss of functions still constitutes a main obstacle in a readily identification of the disease’s traits. As a consequence, a still unmet medical need is to find increasingly reliable functional biomarkers of disease that may bring to an earlier diagnosis and, possibly, predict disease trajectory. To this aim, current research is focusing on cerebrospinal fluid biomarkers and early synaptic alterations. While advances in the research of peripheral biomarkers have been extensively reviewed elsewhere (132), we will focus on the other front with a main question to address: which measurable synaptic changes can be predictive of PD neurodegeneration?

An ideal condition to explore subtle alteration of synaptic activity before neuronal degeneration is offered by animal models of disease in which expression of α-syn is genetically altered. In these models, it is possible to follow the synapse development at different time points along the disease progression and simultaneously study associated motor deficits to find increasingly more sensitive behavioral tests. Both presynaptic and postsynaptic modifications, recently reviewed by Burre, have been associated with α-syn pathology (20). However, given their specific localization at nerve terminals, presynaptic alterations were soon predicted and investigated, leading to seminal papers demonstrating that altered α-syn interferes with SNARE protein assembly, with an associated reduction in exocytosis and DA release (9) thus affecting the activity of the release machinery, although the extent by which α-syn affects neurotransmitter release is debated (20). Relevant to its presynaptic effects, it is noteworthy that α-syn also interacts with other synaptic proteins, such as Synapsin III, a protein that, similar to other members of the synapsin family, plays essential roles in neurotransmitter release, but has an extrasynaptic localization. Interestingly, it has been reported that synapsin III interacts with α-syn in both physiological and pathological conditions, further increasing the complex pattern of presynaptic actions of α-syn pathology (20, 133–136). It seems to be clear, in fact, that due to its ability to mobilize among different sectors of the active zone upon stimulation (137, 138), α-syn dynamic interactions with SNARE, lipidic raft and DAT are highly dependent on the neural activity. Any alteration in this well-tuned machinery is therefore associated with neurotransmission alterations, which may have less impact in basal neurotransmission, but become critical during intense neuronal activity and over their long lifetime. α-syn can also permeabilize lipid membranes through the formation of cations permeable transmembrane pores, thus altering membrane conductances, and increase the risk for altered calcium homeostasis (12, 139).

This effect of α-syn was also studied in cell systems overexpressing the protein (140, 141). Using whole-cell patch-clamp recordings, Feng and coworkers (88) measured ion leakage upon the application of an electrical potential in a dopaminergic cell line. These effects were associated with a modest but significant time-dependent increase in cell death, demonstrating a link between α-syn pathology and conductance changes.

However, while DA release machinery alterations were a primary expected effect of α-syn toxicity, more recent papers have focused on the postsynaptic counterpart of this pathological scenario. Altered activity and distribution of postsynaptic density components have only recently been explored but may be promising tools to detect subtle but measurable changes at the core of this synaptopathy.

Since cognitive alterations have been observed as prodromal PD symptoms early plastic alterations have been first explored in the hippocampus. In 2012, Costa and colleagues studied CA1 hippocampal plasticity in a transgenic mouse model for α-syn aggregation obtained by the expression of human α-syn 120 under the control of the tyrosine hydroxylase promoter (α-syn 120 mice) and leading to the formation of pathological inclusions in the SNc and olfactory bulb and to a reduction in striatal DA levels (142, 143). In a presymptomatic motor stage characterized by spatial memory alterations, CA1 hippocampal pyramidal neurons of α-syn 120 mice show a reduced ability to respond to a high-frequency stimulation with a form of long-lasting plasticity expressed in this area and dependent on DA D1 and NMDA receptors stimulation, called long-term potentiation (LTP). Postsynaptic density modifications were associated with plastic changes as NMDA receptor subunit composition was found changed with a significant decrease of GluN2A/GluN2B subunit ratio. This effect was due to decreased DA release as l-DOPA was able to rescue synaptic functions. Overall, these results first demonstrated that, similar to human condition, cognitive deficit precede motor symptoms with postsynaptic mechanisms (143). In support of this notion, other studies have shown that α-syn plays a role in NMDA receptor trafficking in other brain areas (144–147), suggesting that postsynaptic actions of α-syn impact on intracellular events relevant for synaptic plasticity.

Given the possibility that in pathological conditions α-syn species (monomer, oligomers, and fibrils) may also act extracellularly thus possibly inducing postsynaptic effects, *in vitro* models have been developed to clarify the role of extracellular α-syn in hippocampal plasticity alterations. On this line, the group of Outeiro conducted a series of studies to demonstrate the effects of extracellular α-syn oligomers. The study carried out by Diogenes and colleagues shows that different species of α-syn have distinct effects on synaptic activity. In particular, among oligomers, monomers, and fibrils, only prolonged incubation with oligomers in healthy rodent brain slices were able to increase basal synaptic transmission through a mechanism dependent on NMDA receptor activation, accompanied by an increase in the expression of GluR2-lacking AMPA receptors. In these slices, stimulation with a theta-burst pattern was not able to induce LTP without a previous application of a low-frequency stimulation indicating a saturation effect underlying impairment of LTP (148). Interestingly, these detrimental effects were counteracted by adenosine A2a receptor antagonists, known for their neuroprotective role in PD therapy, and were not observed in animals lacking A2aR. Moreover, blockade of these receptors was able to reduce α-syn aggregates (149).

Another evidence of the link between α-syn overexpression and NMDA receptor dysfunction comes from a recent study of the same group as a further support to the concept that many elements in the postsynaptic compartment play important roles in predisposing the synapse to NMDA-dependent excitotoxicity mediated by its interaction with α-syn aggregates. Using an *in vitro* approach, the authors demonstrated that LTP alterations are caused by the abnormal activity of cellular prion protein, known to act as a cell surface-binding partner for soluble oligomeric protein and to interact with NMDA receptors at postsynaptic density. This protein, when engaged in pathological interactions with α-syn, mediates Ca^2+^ dyshomeostasis and synaptic dysfunction through a mechanism involving Fyn kinase phosphorylation, which is tightly regulated by mGluR5 *via* adenosine A2A receptors. In turn, activated Fyn phosphorylates Y1472 residue of GluN2B-expressing NMDA receptors with consequent excitotoxic effects (150).

Although these studies greatly contributed to the understanding of α-syn synaptic effects, the effects of α-syn oligomers on the functional activity of the striatum have been explored only recently. In fact, the striatum represents the most interesting target for PD therapy since it is the main recipient of dopaminergic nigral neurons, whose activity is impaired by α-syn-mediated toxicity (151). For this reason, transgenic animals overexpressing altered forms of α-syn, such as the truncated human α-syn 1–120 or the WT human α-syn, may be valuable models to assess specific aspects of the pathogenesis of synucleinopathies and to analyze the cell type-specific alterations of striatal synaptic plasticity in the initial phase of the disease.

The first report to show alterations in corticostriatal plasticity associated with α-syn overexpression was provided by an *ex vivo* study performed in slices from mice overexpressing a truncated form of α-syn at a late symptomatic stage of disease (152). But, it was few years later that, taking advantage of the gradual progression of the disease offered by genetic models, Tozzi and coworkers investigated α-syn-mediated alterations in plasticity by studying both spiny projection neurons (SPNs) and cholinergic interneurons (ChIs) in two different models of early PD: the mice transgenic for truncated human α-syn (1–120) and the rat injected with the adeno-associated viral vector carrying WT human α-syn in the SNc (153). In a presymptomatic stage, before any neuronal degeneration, procedural learning deficit was associated in both rodent models with selective impairment of LTP in ChIs but not in SPNs. Similar to what observed in the hippocampus, also here a direct interaction between α-syn and NMDA receptors is suggested, as the loss of LTP in striatal ChIs was dependent on a direct interaction of α-syn with GluN2D-expressing NMDA receptors, which are selectively expressed in this class of interneurons. This link has also been studied in an *in vitro* model. Bath incubation of corticostriatal slices of healthy animals with α-syn oligomers caused the same synaptic alterations that were not rescued by exogenous DA or a D1-like receptor agonist, suggesting that the blockade on synaptic plasticity is not mediated by an α-syn-mediated interference with DA release. These alterations correlate with the behavioral pattern observed, mimicking the early phase of PD, and are in line with what observed in PD patients, in which mild cognitive alterations associated with cholinergic dysfunction, frequently precede overt motor symptoms.

It is noteworthy that micromolar concentration of α-syn oligomers were found effective in determining hippocampal pathology, while nanomolar concentration were sufficient to induce striatal alterations. Taken together, these data suggest that increasing concentrations of α-syn may progressively affect NMDA receptor-mediated functions on distinct neuronal populations, indicating that the vulnerability to this protein may be cell type specific and region specific. On this view, early dysfunction of the striatal cholinergic system, occurring at very low concentrations, represents a possible functional marker of the disease. On the same line of research, a recent study by Giordano and coworkers provided an important link between presynaptic and postsynaptic actions of α-syn and contribute to the reconstruction of a comprehensive view of the many faces of α-syn pathology (154).

Using an animal model of PD, in which animals overexpress human WT α-syn in the midbrain neurons, the authors demonstrate that very early stages are associated with reduced striatal DAT and impaired acquisition of performance plateau in the rotarod task. Interestingly, behavioral impairment has a unique electrophysiological correlate that depends on DAT alteration. In fact, while a form of plasticity, the long-term depression (LTD) is equally expressed at corticostriatal synapses of both WT and α-syn mice before and after exposure to exercise through accelerated rotarod test, healthy animals show an interesting switch from LTD to LTP during the acquisition phase of motor learning. Mice overexpressing human α-syn do not show this shift in plasticity that is instrumental to acquire motor habits and perform correctly. This early training-induced shift from LTD to LTP, and the achievement of a good performance, is impaired in control animals pretreated with DAT inhibitor GBR-12909. These findings, in line with previous studies (155–159), further suggest that early signs of synucleinopathy do not necessarily correlate with DA neuronal loss and support the notion that a reorganization of cellular plasticity within the dorsal striatum is necessary for the acquisition of a motor skill, and it depends on an intact dopaminergic transmission, controlled by DAT, which is impaired early by nigral overexpression of human α-syn.

## Conclusion

**Figure 1:**
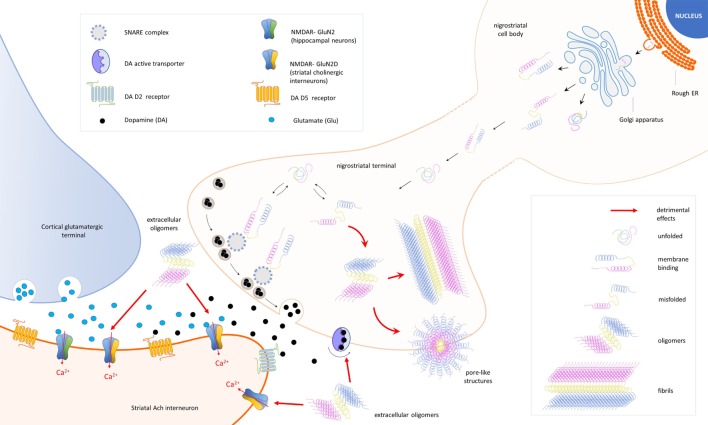
Schematic representation of the cellular and synaptic detrimental actions mediated by different forms of the protein alpha-synuclein (α-syn). In nigral neurons, endoplasmic reticulum (rough ER), *SNCA* transcripts are translated into native α-syn proteins, which are assembled in the Golgi apparatus and released in different conformations. Due to its auto-chaperone activity, α-syn exists in a dynamic balance between monomeric unfolded and amphipathic alpha-helix (membrane binding) state, adopting a range of conformations depending on the environment and binding partners. During the assembly process, misfolding proteins might be also produced (misfolded) and escape detection and clearance by intracellular quality control systems. After synapse maturation, α-syn migrates to nerve terminals and interacts with intracellular proteins [SNAP REceptor (SNARE) complex] and the dopamine (DA) active trasporter to ensure a correct control of neurotransmission. Misfolded α-syn may combine into oligomers that, under specific stimulations, form transmembrane pore-like structures able to alter membrane conductances. Overexpression of α-syn exacerbates pathological events and culminates with the formation of fibrillar aggregates (fibrils), a major component of Lewy bodies. Extracellular α-syn oligomers interfere with the expression of long-term potentiation, a form of synaptic plasticity mediated by *N*-methyl-d-aspartate receptors (NMDAR) in striatal cholinergic interneurons. A direct interaction between α-syn and the GluN2D subunit has been demonstrated in three different models of experimental parkinsonism.

Taken together, all these findings return a complex but increasingly clear scenario designed by the many roles of α-syn (Figure 1; Table 1).

**Table 1:** Summary of the findings on the role of α-syn in the distinct aspects contributing to pathogenesis of Parkinson’s disease (PD).

	Reference	Findings	Experimental conditions
Neurodegeneration	(22)	*C-terminally truncated alpha-synuclein* (α-syn), particularly α-syn (1–120), assembles into filaments morphologically very similar to those seen in neurodegenerative conditions	Ala^30^Pro α-syn, Ala^53^Thr α-syn, α-syn (1–110), α-syn (1–120), and α-syn (1–130) subcloned into the bacterial expression vector pRK172 and expressed in *Escherichia coli* BL21(DE3)

	(52)	α-syn lacking residues 71–82 (*α-synΔ71–82*) are unable to aggregate and show no dopaminergic neurotoxicity, whereas truncated C-terminal α-syn (*α-synΔ120*) has a moderate role in influencing both aggregation and toxicity	Transgenic *Drosophila* modified to obtain α-synΔ71–82 or α-synΔ120

	(40)	*Nigrostriatal α-syn* levels increase with age causing inclusion bodies to form in nigral neurons and drive dopamine (DA) levels over a symptomatic threshold	Human and non-human primate models

	(41)	*A53T* α-syn, but not ΔDQ/A53T, causes toxicity in primary cortical neurons through chaperone activity dysfunction and aberrant macroautophagy activation	Stable rat PC12 and human SH-SY5Y cells inducibly expressing human wild-type (WT) α-syn, ΔDQ/WT α-syn, A53T α-syn, and ΔDQ/A53T α-syn

	(53)	Co-expression of human full-length α-syn (*αsynFL*) and C-terminally truncated human α-syn (*αsynΔ110*) can augment the accumulation of pathological αsynFL protein and lead to dopaminergic cell death	Adult Sprague-Dawley rats injected in the SN with viral vector rAAV5-asynFL + rAAV5-asynD110

	(42)	WT α-syn overexpression causes a decrease in LC3-II levels impairing autophagy which increases accumulation of aggregate-prone proteins and sensitizes the cell to proapoptotic assaults	Human neuroblastoma cells (SKNSH), human cervical carcinoma cells (HeLa), and human embryonic kidney cells (HEK293)

	(43)	The introduction of small amounts of pre-formed *α-syn fibrils* into α-syn-expressing cells results in aggregation of endogenously expressed α-syn and the formation of insoluble aggregates which persist even when soluble α-syn levels are substantially reduced, indicating their refractoriness to clearance	Mammalian and primary neuronal cell cultures—HEK293 cells stably expressing WT or A53T α-syn

	(88)	*α-syn overexpression* results in increased oligomer production and formation of membrane-bound α-syn-containing pores that induce increase in membrane conductance, determining cell death	MN9DwtsynIR-Esgfp (MN9Dsyn) cells were derived and engineered from mouse embryonic mesencephalon

	(13)	Penetration of α-syn into membranes gives rise to the formation of *annular pore-like oligomer structures* with the ability to increase cell permeability and calcium influx	Cell culture of neuronal cells expressing WT or mutant A53T α-syn

	(86)	α-syn activates *calcineurin* (*CN*), mediating the translocation of NFATc3, which contributes to the loss of neurons. Administration of CN inhibitor cyclosporine A rescues the α-syn-induced loss of primary mDA neuron cultures	HEK293 cells transfected with WT and A53T α-syn cDNAs

	(98)	Exogenous α-syn strains seed the assembly of endogenous α-syn. Differently from α-syn *oligomers*, α-syn *fibrils* and *ribbons* remain in place after crossing the blood–brain barrier. Distinct α-syn assemblies can affect neurotransmission after acute exposure, but only fibrillar α-syn exhibits perpetual behavioral and aggravated neurotoxic phenotypes *in vivo*	Intracerebral injections of exogenous α-syn in Wistar rats

Oxidative stress	(48)	*Nitration of Tyr residues* appears to prevent fibrillogenesis from soluble α-syn proteins. Oxidative stress causes soluble α-syn to form covalently linked dimers and higher oligomers and allows for fibril formation and stabilization	WT and Tyr to Phe mutant recombinant human α-syn proteins expressed in *E. coli* BL21(DE3) RIL cells

	(49)	*Nitration* effectively inhibits fibrillation of α-syn. The addition of low concentration of nitrated α-syn inhibits the fibrillation of non-modified α-syn	Human WT α-syn was expressed using *E. coli* BL21

Immune response	(126)	Autoimmune response to α-syn can serve as a valid *biomarker*, reflecting the progressive brain neurodegeneration and impaired α-syn homeostasis occurring in PD	Analysis of human PD patients’ serum

	(91)	Changes in human microbiota are correlated to the motor and *gastrointestinal parkinsonian dysfunctions* by impacting neuroinflammation and α-syn aggregation. *Gut bacteria* from PD patients promote enhanced motor impairment when transplanted into α-syn overexpressing mice	Germ free male α-syn overexpressing and WT colonized with fecal microbes from PD patients and healthy controls

	(131)	Peptides derived from two regions of α-syn (Y39 and S129) produce immune responses in patients with PD which are enacted mostly by IL-5-secreting CD4^+^ T cells, as well as IFNγ -secreting CD8^+^ cytotoxic T cells. The Y39 antigenic region is strikingly close to the α-syn mutations that cause PD	Genome sequencing of PD patients

Synaptic alterations	(23)	The *double α/β synuclein deletion*, but not single ones, decreases DA levels, and impairs synaptic parameters (structure of synapse, neurotransmitters release, mobilization of synaptic vesicles, or forms of short- and long-term synaptic plasticity)	α^+/+^ β^+/+^, α^−/−^ β^+/+^, α^+/+^ β^−/−^, α^−/−^ β^−/−^-syn mice

	(25)	In aged *α-syn null mice* a dramatic effect on synapse structure, a decrease conduction velocity and a lower neuronal excitability are observed. Modest overexpression of human α-syn in young mice causes a decrement in neurotransmission similar to aged α-syn null mice	αβγ-syn triple KO mice

	(63)	In dopaminergic neurons, intracellular α-syn induces a DA active trasporter *(DAT)-mediated inward current* extracellular Na^+^ independent but transmembrane Cl^−^ gradient sensitive, which is eliminated by DAT antagonist GBR12935 and is absent with intracellular heat-inactivated α-syn. These changes are paralleled by an α-syn-dependent decrease in rate of DAT-mediated substrate uptake. The membrane potential of cells overexpressing α-syn rest at more depolarized state, disrupting cell homeostasis	Primary neuronal culture of acutely dissociated TH:RFP mouse midbrain DA neurons

	(64)	DA uptake and DAT distribution in striatal membranes are dysregulated in *young mice* overexpressing A53T α-syn. Uptake of DA through DAT is normalized in *older animals* where bioavailability of *A53T α-syn* is reduced and expression of β-syn and γ-syn increased. The normalization of DA uptake with aging may relate to a shift in modulation of DAT from α-syn to other synucleins	Transgenic mice expressing mutant A53T α-syn

	(153)	Overexpression of either truncated or full-length human *α-syn affects plasticity of* cholinergic interneurons (*ChIs*), but not of spiny projection neurons, resulting in mild cognitive and motor deficits, mimicking early PD. Acute application of exogenous human α-syn oligomers to striatal slices of control animals impairs long-term potentiation of ChIs by targeting *GluN2D*-expressing *N*-methyl-d-aspartate receptors (NMDARs). Subchronic l-DOPA restores synaptic plasticity in ChIs, suggesting that a long-term dopaminergic activation is required to compensate for the complex molecular effects induced by DA denervation on NMDAR subunits	Transgenic mice expressing truncated human α-syn (1–120) and Sprague-Dawley rats injected with the adeno-associated viral vector (AAV) carrying WT human α-syn (AAV-α-syn)

	(154)	Early versus optimal motor learning changes *striatal DAT levels*. DAT-regulated activation of the D1 pathway in the dorsolateral striatum, during early-stage of incremental motor learning, cooperates with D1 pathway activation to prevent premature shifting to habit learning. Overexpression of α-syn impairs motor learning by altering DAT expression, before leading to DA neuronal loss and bradykinesia	CD1 mice model of PD by performing bilateral injections of recombinant adeno-associated viral vector (rAAV)-hu-α-syn and rAAVGFP in the SNpc/VTA (ventral tegmental area)

Future studies will be needed to further investigate how endogenous molecules interact with different α-syn conformations. In particular, based on the majority of data reviewed here, we expect that more effort will be aimed at explaining the mechanisms underlying distinct cell-type and region-specific α-syn-mediated NMDA receptor alterations. Moreover, in light of the latter study and of many reports supporting the neuroprotective effect of intense exercise in newly designed rehabilitation programs (160, 161), a promising link to explore will be the interaction between α-syn aggregation and experience.

## Author Contributions

VG wrote and revised the manuscript, prepared the figure and reviewed the final version; VC revised the manuscript, prepared the review table and proofread the final version; PC conceived and designed the manuscript, edited, and proofread the final version of the paper.

## Conflict of Interest Statement

The authors declare that the research was conducted in the absence of any commercial or financial relationships that could be construed as a potential conflict of interest.
